# Poisons and Poisoners,—II

**Published:** 1891-04-11

**Authors:** 


### Poisons and Poisoners.—II.

### I.—ARSENIC: MADELINE SMITH (*concluded*).

Madeline had bought arsenic three times. This was Miss Smith's third purchase of arsenic. She had bought some in February; and on the 19th of that month L'Angelier, coming home late at night to his lodgings, suffered from what his landlady thought was a bilious attack. He, however, declared that he was not subject to biliousness, and certainly this attack left more permanent effects than in common with such ailments. Indeed, L'Angelier never really recovered from it. He had, it seems, mentioned to a Miss Perry, who knew of the attachment between him and Miss Smith, that he expected to meet Madeline on the evening of the 19th, but it could not be distinctly proved that the meeting took place. He also told Miss Perry that Madeline had given him a cup of coffee and chocolate, and added, "I can't think why I was so unwell after getting that coffee and chocolate from her."

After this Madeline changed her tone. On March 5th L'Angelier wrote to her demanding a distinct reply to the rumours he had heard of her engagement to Mr. Minnoch. On the 6th she bought more arsenic, again making an excuse about rats. On the 10th she went to Bridge of Allan, a watering-place not far from Stirling, with her family, and from there she wrote to him in the following terms: "I think we shall be home on Tuesday, so I shall let you know, my own beloved, sweet pet, when we shall have a dear, sweet interview; when I may be pressed to your heart, and kissed by you, my own sweet love. A fond, tender embrace; a kiss, sweet love!"

Of such language, written by any woman to any man, the best one can say would be in the words of the carpenter in "Through the Looking-glass," "the butter's spread too thick." But the woman who wrote this had already fixed the day for her marriage with another man. Her words are either hypocritical or shameless. In any case they are revolting. On March 18th, having returned to Glasgow, Miss Smith purchased arsenic for the third time. She also wrote to L'Angelier, making an appointment with him for the 19th. But by this time he, having obtained a holiday on the plea of ill-health, had gone to Bridge of Allan, only to find the Smith family gone. On the 20th she wrote again: "Why, my beloved, did you not come to me? Oh, my beloved, are you ill? Come to me. Sweet one, I waited and waited for you, but you came not. I shall wait again to-morrow night—same hour and arrangement." This letter was forwarded to L'Angelier by a fellow lodger, and immediately on receipt of it he returned to Glasgow. The appointment was for Saturday, the 21st of March, but L'Angelier did not receive the note till Sunday, the 22nd. He reached his lodgings in Glasgow at eight o'clock on the evening of that day, and immediately went out. He did not return till two o'clock in the morning, when he rang the bell violently, and his landlady on opening the door found him evidently in intense pain. The attack was in all its symptoms like that from which he had suffered the previous month, and in a few hours he was dead.

Medical aid had been summoned, but the doctor's efforts had been directed solely to relieving the pain, and it was not till other circumstances had aroused suspicion that L'Angelier's body was exhumed and examined, when there was found in it enough arsenic to leave no doubt as to the manner of his death. Who administered the poison? Only one person in the world had cause to wish Emile L'Angelier's death; that person was Madeline Smith. His fellow countryman, De Meau, to whom L'Angelier had said, a few weeks before, that if it was true Miss Smith thought of marrying Mr. Minnoch, "he had documents in his possession that would be sufficient to forbid the banns," called on the evening of L'Angelier's death on Mr. Smith, and advised him, for the sake of his daughter's reputation, to try to gain possession of these documents. He also saw Miss Smith in her mother's presence, and questioned her as to when she had last seen L'Angelier. The girl admitted the assignation for the 21st, but denied that the meeting that had failed to come off that night had taken place on the 22nd. On the contrary, she protested that she had not seen him for three weeks. There can be no doubt that the impossibility of settling this question saved her life, though another point may have had some weight with the jury, which was subsequently proved by Sir Robert Christison to be valueless. This was that so heavy and insoluble a substance as the arsenic Miss Smith bought could not have bean administered in a cup of cocoa—the suspected vehicle—without attracting the attention of the person who drank it. But Dr. Christison met with a case in which arsenic had been administered in hot whiskey and water without the suspicion of the victim, the arsenic being kept in suspension till the moment of drinking by constant stirring. But the chief argument in favour of the accused, the one which cast on the whole case sufficient doubt to save her, was the fact that she had not made an appointment with L'Angelier for the night on which he was poisoned, and that apparently no human creature had seen them meet. The charge of murder was not indeed "disproved," but, as the jury decided—in a Scotch jury of fifteen the vote of the majority rules — "not proven," and Madeline Smith escaped.

It is not for us to express a more decided opinion than that of the Court which tried her; but on one point we may fitly comment. This young girl could, by giving some flimsy excuse about rats, obtain enough arsenic to poison any man. She had to sign her name in a book; that was the only formality required. Possibly, if she had chosen to give a false name and address, it would have made little difference. Surely this is too facile. With only the slightest trouble anyone may become possessed of a supply of deadly poison. The signature in the book may help to trace a criminal now and then, but it is a very poor protection to the destined victim. It is surely not too much to ask that no poison should be sold without a prescription from a medical man. Some of these have, indeed, abused their privileges for evil ends, but in these cases there was the bad excuse of selfish interests to serve; and we doubt if even the worst of them would authorise a layman to obtain poison without knowing exactly for what it is to be used. The present restrictions are too feeble, and with our opportunities for obtaining so many deadly drugs, both under their own names and as components of so many patent medicines, it says something for the moral sense of the community that the privilege is not more abused. But it is possible that there are many murders committed by means of poison that never come to light, and it may be feared that it is the dread of detection that keeps many people from venturing on the dangerous path of crime. At least, some such impression is conveyed by the following anecdote: In a trial for murder by poison that took place some twelve years ago one of the medical witnesses was asked if there was any poison which it was practically impossible to trace. He answered that in his opinion there was just one. "Then, sir," said the presiding Judge, "I will ask you *not* to mention its name." The next day the doctor was besieged with letters inquiring the name of the drug. All the writers professed to be moved only by an impersonal interest in science. But the doctor did not gratify their curiosity. *Verbum sap.*

